# Supplementary material for: Diagnostic and prognostic potential of eight whole blood microRNAs for equine sarcoid disease
Source: PLoS One. 2021 Dec 23;16(12):e0261076. doi: 10.1371/journal.pone.0261076 (PMC8699634; doi:10.1371/journal.pone.0261076)
Supplement: S1 Table — (DOCX) [file pone.0261076.s001.docx]

**Supplementary Table 1: TaqMan MicroRNA Assay kits used for RT-qPCR**

| **miRNA** | **Company** | **TaqMan™ MicroRNA Assay Name** | **Catalogue Number** | **Assay ID** |
| --- | --- | --- | --- | --- |
| eca-miR-24 | ThermoFisher, Reinach, Switzerland | hsa-miR-24-3p | 4427975 | 477992_mir |
| eca-miR-107b |  | hsa-miR-107 |  | 478254_mir |
| eca-miR-125a-5p |  | hsa-miR-125a-5p |  | 477884_mir |
| eca-miR-127 |  | hsa-miR-127-3p |  | 477889_mir |
| eca-miR-134 |  | hsa-miR-134-5p |  | 477901_mir |
| eca-miR-323-5p |  | hsa-miR-323a-5p |  | 4778824_mir |
| eca-miR-379 |  | hsa-miR-379-5p |  | 478077_mir |
| eca-miR-381 |  | hsa-miR-381-3p |  | 477816_mir |
| eca-miR-382 |  | hsa-miR-382-5p |  | 478078_mir |
| eca-miR-432 |  | hsa-miR-432-5p |  | 478101_mir |
| eca-miR-30d |  | hsa-miR-30d-5p |  | 478606_mir |
| cel-miR-39-3p |  | cel-miR-39-3p |  | 478293_mir |
