# Supplementary material for: Diagnostic and prognostic potential of eight whole blood microRNAs for equine sarcoid disease
Source: PLoS One. 2021 Dec 23;16(12):e0261076. doi: 10.1371/journal.pone.0261076 (PMC8699634; doi:10.1371/journal.pone.0261076)
Supplement: S2 Table — All horses were three years old at the time of initial examination. Information on biological variables (breed, sex) and time between initial and follow-up examination is provided. For ES affected horses, information on worst ES type as well as localization and number of ES lesions at both examinations and on the type of therapy applied is given. Abbreviations: ES = Equine sacoid, ID = identity, FM = Franches-Montagnes, WB = Swiss Warmblood, NA = not applicable, f = female, m = male, mc = male castrated. (DOCX) [file pone.0261076.s002.docx]

**Supplementary Table 2: Overview study cohort**. All horses were three years old at the time of the first examination. Information on biological variables (breed, sex) and time between the first and second examination is provided. For ES-affected horses information on worst ES type as well as localization and number of ES lesions at both examinations and on the type of therapy applied is given, if available.

| **Clinical course** | **ID** | **breed** | **sex** | **1^st^ examination of sarcoids** | | | **Time between examina-tions** | **2^nd^ examination of sarcoids** | | | **type of therapy** |
| --- | --- | --- | --- | --- | --- | --- | --- | --- | --- | --- | --- |
|  |  |  |  | **Worst type** | **Localization** | **Number** |  | **Worst type** | **Localization** | **Number** |  |
| **Regression** | 1602 | FM | f | occult | ventrum | 1 | 5 | NA | NA | 0 | none |
| **Regression** | 1648 | FM | f | verrucous | head/ ventrum | 2 | 5 | NA | NA | 0 | none |
| **Regression** | 1596 | FM | mc | verrucous | inguinal/ ventrum | 2 | 5 | NA | NA | 0 | none |
| **Regression** | 1609 | FM | f | unknown | ventrum | 1 | 5 | NA | NA | 0 | none |
| **Regression** | 1610 | FM | f | occult | ventrum | 1 | 5 | NA | NA | 0 | none |
| **Regression** | 1645 | FM | mc | occult | ventrum | 1 | 5 | NA | NA | 0 | none |
| **Regression** | 1646 | FM | f | unknown | inguinal | 2 | 5 | NA | NA | 0 | none |
| **Regression** | 1659 | FM | f | occult | ventrum | 1 | 5 | NA | NA | 0 | none |
| **Regression** | 1653 | FM | f | verrucous | head/ ventrum | 2 | 5 | NA | NA | 0 | none |
| **Regression** | 071053 | WB | f | not reported | chest | 2 | 9 | NA | NA | 0 | none |
| **Regression** | 060404 | WB | f | not reported | chest | 1 | 10 | NA | NA | 0 | none |
| **Regression** | 050309 | WB | mc | not reported | chest | 3 | 11 | NA | NA | 0 | none |
| **Regression** | 060317 | WB | f | not reported | ventrum | 1 | 11 | NA | NA | 0 | none |
| **Regression** | 060583 | WB | f | not reported | ventrum | 1 | 10 | NA | NA | 0 | none |
| **Regression** | 070975 | WB | mc | not reported | not reported | 1 | 9 | NA | NA | 0 | unknown |
| **Regression** | 070966 | WB | mc | not reported | chest | 1 | 9 | NA | NA | 0 | excision |
| **Regression** | 060547 | WB | mc | not reported | inguinal/ ventrum | 2 | 10 | NA | NA | 0 | none |
| **Regression** | 050130 | WB | f | not reported | chest | 1 | 12 | NA | NA | 0 | immunotherapy |
| **Regression** | 050441 | WB | mc | not reported | limb | 1 | 11 | NA | NA | 0 | none |
| **Progression** | 040265 | FM | mc | occult | head/ inguinal | 2 | 5 | verrucous | axilla/ neck | 2 | Excision/ AM |
| **Progression** | 040082 | FM | mc | occult | ventrum | >5 | 7 | nodular | chest/ prepuce | 2 | unknown ointment/ excision |
| **Progression** | 040455 | FM | f | verrucous | inguinal/ ventrum | 2 | 5 | verrucous | others | 2^[[1]](#endnote-1)^ | none |
| **Progression** | 070625 | WB | f | not reported | not reported | >5 | none^[[2]](#endnote-2)^ | unknown | multiple | >5 | multiple therapies |
| **Progression** | 120017 | WB | f | verrucous | ventrum | 1 | 6 | fibroblastic | inguinal/ udder/ ventrum | >5 | AM/ X-Terra |
| **Progression** | 071002 | WB | f | not reported | not reported | 3 | 9 | verrucous | chest/ limb/ ventrum | >5 | none |
| **Progression** | 130452 | WB | mc | verrucous | chest/ neck | >5 | 5 | mixed | multiple | >5 | AM/ mistletoe extract |
| **Progression** | 080400 | WB | mc | nodular | chest/ head/ neck | 1 | 10 | mixed | multiple | >5 | multiple |
| **Progression** | 060431 | WB | mc | not reported | head | 2 | 10 | nodular | chest/ inguinal/ ventrum | >5 | none |
| **New occurrence** | 040077 | FM | f | NA | NA | 0 | 5 | verrucous | ventrum | 1 | none |
| **New occurrence** | 091122 | FM | f | NA | NA | 0 | 5 | verrucous | ventrum | 1 | none |
| **New occurrence** | 040090 | FM | mc | NA | NA | 0 | 5 | verrucous | limbs | 1 | AM |
| **New occurrence** | 040682 | FM | f | NA | NA | 0 | 5 | nodular | chest/ head/ inguinal | >5 | none |
| **New occurrence** | 100327 | FM | f | NA | NA | 0 | 5 | mixed | chest/ inguinal/ ventrum | >5 | none |
| **New occurrence** | 070509 | WB | f | NA | NA | 0 | 10 | mixed | chest/ inguinal | >5 | none |
| **New occurrence** | 071040 | WB | mc | NA | NA | 0 | 9 | nodular | axilla/ head/ inguinal | >5 | excision/ chemotherapy/ AM |
| **New occurrence** | 060578 | WB | mc | NA | NA | 0 | 10 | nodular | limb/ ventrum | 2 | chemotherapy |
| **New occurrence** | 060561 | WB | mc | NA | NA | 0 | 10 | fibroblastic | chest/ inguinal/ ventrum | 3 | ligation/ immunotherapy |
| **New occurrence** | 070822 | WB | mc | NA | NA | 0 | 9 | mixed | inguinal/ limb | 2 | none |
| **New occurrence** | 050458 | WB | f | NA | NA | 0 | 11 | verrucous | inguinal/ ventrum | 2 | none |
| **New occurrence** | 070499 | WB | mc | NA | NA | 0 | 10 | mixed | chest/ limb/ ventrum | >5 | AM |
| **New occurrence** | 060469 | WB | mc | NA | NA | 0 | 10 | mixed | axilla/ chest/ head | >5 | chemotherapy/ immunotherapy/ excision/ AM |
| **New occurrence** | 060342 | WB | f | NA | NA | 0 | 11 | fibroblastic | head/ inguinal/ limb | 3 | chemotherapy/ AM |
| **New occurrence** | 040876 | WB | mc | NA | NA | 0 | 12 | nodular | chest/ Inguinal/ limb | >5 | AM |
| **New occurrence** | 070947 | WB | mc | NA | NA | 0 | 9 | nodular | chest/ inguinal/ ventrum | 3 | chemotherapy |
| **New occurrence** | 071005 | WB | mc | NA | NA | 0 | 10 | nodular | inguinal/ ventrum | 3 | ointment/ excision/ homeopathy |
| **New occurrence** | 060525 | WB | mc | NA | NA | 0 | 11 | verrucous | head/ inguinal/ ventrum | >5 | unknown ointment |
| **New occurrence** | 060598 | WB | mc | NA | NA | 0 | 10 | nodular | axilla/ inguinal | >5 | ligation |
| **Control** | 040813 | FM | m | NA | NA | 0 | 5 | NA | NA | 0 | none |
| **Control** | 092132 | FM | f | NA | NA | 0 | 5 | NA | NA | 0 | none |
| **Control** | 040218 | FM | f | NA | NA | 0 | 5 | NA | NA | 0 | none |
| **Control** | 040553 | FM | f | NA | NA | 0 | 5 | NA | NA | 0 | none |
| **Control** | 040717 | FM | f | NA | NA | 0 | 5 | NA | NA | 0 | none |
| **Control** | 091913 | FM | f | NA | NA | 0 | 5 | NA | NA | 0 | none |
| **Control** | 091108 | FM | f | NA | NA | 0 | 5 | NA | NA | 0 | none |
| **Control** | 091378 | FM | f | NA | NA | 0 | 5 | NA | NA | 0 | none |
| **Control** | 040217 | FM | f | NA | NA | 0 | 5 | NA | NA | 0 | none |
| **Control** | 040564 | FM | f | NA | NA | 0 | 5 | NA | NA | 0 | none |
| **Control** | 040753 | FM | mc | NA | NA | 0 | 5 | NA | NA | 0 | none |
| **Control** | 040261 | FM | mc | NA | NA | 0 | 6 | NA | NA | 0 | none |
| **Control** | 040487 | FM | mc | NA | NA | 0 | 6 | NA | NA | 0 | none |
| **Control** | 040126 | FM | mc | NA | NA | 0 | 6 | NA | NA | 0 | none |
| **Control** | 040304 | FM | mc | NA | NA | 0 | 6 | NA | NA | 0 | none |
| **Control** | 060281 | WB | f | NA | NA | 0 | 11 | NA | NA | 0 | none |
| **Control** | 060320 | WB | f | NA | NA | 0 | 11 | NA | NA | 0 | none |
| **Control** | 071011 | WB | mc | NA | NA | 0 | 9 | NA | NA | 0 | none |
| **Control** | 060348 | WB | f | NA | NA | 0 | 11 | NA | NA | 0 | none |
| **Control** | 070979 | WB | f | NA | NA | 0 | 9 | NA | NA | 0 | none |
| **Control** | 050275 | WB | f | NA | NA | 0 | 12 | NA | NA | 0 | none |
| **Control** | 050206 | WB | f | NA | NA | 0 | 12 | NA | NA | 0 | none |
| **Control** | 050104 | WB | f | NA | NA | 0 | 12 | NA | NA | 0 | none |
| **Control** | 050550 | WB | mc | NA | NA | 0 | 11 | NA | NA | 0 | none |
| **Control** | 070501 | WB | f | NA | NA | 0 | 10 | NA | NA | 0 | none |
| **Control** | 060429 | WB | mc | NA | NA | 0 | 11 | NA | NA | 0 | none |
| **Control** | 060512 | WB | mc | NA | NA | 0 | 11 | NA | NA | 0 | none |
| **Control** | 050628 | WB | mc | NA | NA | 0 | 12 | NA | NA | 0 | none |
| **Control** | 070951 | WB | m | NA | NA | 0 | 10 | NA | NA | 0 | none |
| **Control** | 050490 | WB | mc | NA | NA | 0 | 12 | NA | NA | 0 | none |

f: female

m: male

mc: male castrated

FM: Franches-Montagnes

WB: Swiss Warmblood

NA: not applicable

AM: alternative medicine

RGR: regression

PGR: progression

NO: new occurrence

CTL: control

1. Increased in size [↑](#endnote-ref-1)
2. Was euthanized due to PGR of ES-lesions, 2^nd^ examination performed by a veterinarian in the field [↑](#endnote-ref-2)
