# Supplementary material for: Diagnostic and prognostic potential of eight whole blood microRNAs for equine sarcoid disease
Source: PLoS One. 2021 Dec 23;16(12):e0261076. doi: 10.1371/journal.pone.0261076 (PMC8699634; doi:10.1371/journal.pone.0261076)
Supplement: S5 Table — Abbreviations: Cq = quantification cycle, CI = Confidence interval. (DOCX) [file pone.0261076.s005.docx]

**Supplementary Table 5**: Pearson correlation coefficients between the two normalization methods used in this study for each miRNA candidate and control with 95% Confidence interval (CI)

| **Candidate** | **Pearson Correlation (CI)** |
| --- | --- |
| Eca-miR-24 | 0.92 (0.87- 0.95) |
| Eca-miR-125a-5p | 0.99 (0.99- 1.00) |
| Eca-miR-127 | 0.993 (0.988- 0.995) |
| Eca-miR-134 | 0.97 (0.96- 0.98) |
| Eca-miR-379 | 0.996 (0.994- 0.997) |
| Eca-miR-381 | 0.9992 (0.9987- 0.9995) |
| Eca-miR-382 | 0.997 (0.996- 0.998) |
| Eca-miR-432 | 0.992 (0.987- 0.995) |
