# Supplementary material for: Diagnostic and prognostic potential of eight whole blood microRNAs for equine sarcoid disease
Source: PLoS One. 2021 Dec 23;16(12):e0261076. doi: 10.1371/journal.pone.0261076 (PMC8699634; doi:10.1371/journal.pone.0261076)
Supplement: S6 Table — The first model (A) assessed the diagnostic potential of the candidate miRNAs, the second model (B) evaluated the potential of miRNA to predict the outcome in horses with ES-lesions and the third model (C) examined the prognostic potential of the candidate miRNAs to predict new appearance of ES. (DOCX) [file pone.0261076.s006.docx]

**Table 2: Results of the MANOVA Analysis for the 3 Models**

| A - First model: evaluation of diagnostic potential of the candidate miRNAs (CLT + NO vs. RGR + PGR) | | | | | | | | | | | | | | |
| --- | --- | --- | --- | --- | --- | --- | --- | --- | --- | --- | --- | --- | --- | --- |
| **2^-∆∆Cq^ method** | | | | | | | | | | | | | | |
|  | Sex | | | | Breed | | | Diagnosis | | | Sex + diagnosis | Breed+ diagnosis | | |
| Multivariate analysis | 0.02 | | | | 0.002 | | | <0.0001 | | | 0.21 | 0.002 | | |
| univariate analysis | | | | | | | | | | | | | | |
|  | p | p adj | | sig | p | p adj | sig | p | p adj | sig |  | p | p adj | sig |
| eca-miR-24 | 0.45 |  | |  | 0.55 |  |  | 0.52 |  |  |  | 0.82 |  |  |
| eca-miR-125a-5p | 0.64 |  | |  | 0.42 |  |  | 0.09 |  |  |  | 0.24 |  |  |
| eca-miR-127 | <0.0001 | 0.006 | | Yes | 0.48 |  |  | 0.002 | 0.006 | Yes |  | 0.0009 | 0.006 | Yes |
| eca-miR-134 | 0.002 | 0.03 | | Yes | 0.014 | 0.018 | Yes | 0.04 | 0.01 | No |  | 0.17 |  |  |
| eca-miR-379 | 0.0004 | 0.02 | | Yes | 0.5 |  |  | 0.04 | 0.02 | No |  | 0.49 |  |  |
| eca-miR-381 | 0.28 |  | |  | 0.0128 | 0.0125 | No | 0.49 |  |  |  | 0.38 |  |  |
| eca-miR-382 | 0.0009 | 0.03 | | Yes | 0.002 | 0.006 | Yes | 0.58 |  |  |  | 0.37 |  |  |
| eca-miR-432 | 0.0002 | 0.01 | | Yes | 0.10 |  |  | 0.18 |  |  |  | 0.07 |  |  |
| **Normalized copy numbers method** | | | | | | | | | | | | | | |
|  | Sex | | | | Breed | | | Diagnosis | | | Sex + diagnosis | Breed+ diagnosis | | |
| Multivariate analysis | 0.02 | | | | 0.0005 | | | < 0.0001 | | | 0.09 | 0.00003 | | |
| univariate analysis | | | | | | | | | | | | | | |
|  | p | p adj | | sig | p | p adj | sig | p | p adj | sig |  | p | p adj | sig |
| eca-miR-24 | 0.0002 | 0.01 | | Yes | 0.68 |  |  | 0.0008 | 0.006 | Yes |  | 0.98 |  |  |
| eca-miR-125a-5p | 0.0003 | 0.02 | | Yes | 0.34 |  |  | 0.03 | 0.019 | No |  | 0.18 |  |  |
| eca-miR-127 | 0.0006 | 0.02 | | Yes | 0.0007 | 0.006 | Yes | 0.05 | 0.01 | No |  | 0.001 | 0.006 | Yes |
| eca-miR-134 | 0.63 |  | |  | 0.39 |  |  | 0.09 |  |  |  | 0.2 |  |  |
| eca-miR-379 | 0.25 |  | |  | 0.008 | 0.02 | Yes | 0.15 |  |  |  | 0.36 |  |  |
| eca-miR-381 | 0.0008 | 0.03 | | Yes | 0.001 | 0.01 | Yes | 0.52 |  |  |  | 0.32 |  |  |
| eca-miR-382 | <0.0001 | 0.006 | | Yes | 0.05 |  |  | 0.15 |  |  |  | 0.35 |  |  |
| eca-miR-432 | 0.33 |  | |  | 0.06 |  |  | 0.35 |  |  |  | 0.051 |  |  |
| B - Second model : evaluation of the prognostic potential of the candidate miRNAs for the development of ES lesions in ES-affected horses (NO vs. CTL) | | |  |  |  |  |  |  |  |  |  |  |  |  |
| **2^-∆∆Cq^ method** | | |  |  |  |  |  |  |  |  |  |  |  |  |
|  | Prognosis | |  |  |  |  |  |  |  |  |  |  |  |  |
| Multivariate analysis | 0.78 | |  |  |  |  |  |  |  |  |  |  |  |  |
| **Normalized copy number method** | | |  |  |  |  |  |  |  |  |  |  |  |  |
|  | Prognosis | |  |  |  |  |  |  |  |  |  |  |  |  |
| Multivariate analysis | 0.75 | |  |  |  |  |  |  |  |  |  |  |  |  |

| C – Third model: evaluation of the prognostic potential of the candidate miRNAs for the prediction of new appearance of ES lesions in so far ES-free horses (NO vs. CTL) | | | | | | | | |
| --- | --- | --- | --- | --- | --- | --- | --- | --- |
| **2^-∆∆Cq^ method** | | | | | | | | |
|  | Sex | | | Prognosis | Sex + prognosis | | | Breed+ prognosis |
| Multivariate analysis | 0.04 | | | 0.06 | 0.005 | | | 0.98 |
| univariate analysis | | | | | | | | |
|  | p | p adj | sig |  | p | p adj | sig |  |
| eca-miR-24 | 0.81 |  |  |  | 0.19 |  |  |  |
| eca-miR-125a-5p | 0.51 |  |  |  | 0.02 | 0.03 | Yes |  |
| eca-miR-127 | 0.0006 | 0.0062 | Yes |  | 0.002 | 0.01 | Yes |  |
| eca-miR-134 | 0.005 | 0.01 | Yes |  | 0.07 |  |  |  |
| eca-miR-379 | 0.006 | 0.03 | Yes |  | 0.001 | 0.006 | Yes |  |
| eca-miR-381 | 0.90 |  |  |  | 0.38 |  |  |  |
| eca-miR-382 | 0.009 | 0.03 | Yes |  | 0.07 |  |  |  |
| eca-miR-432 | 0.002 | 0.01 | Yes |  | 0.002 | 0.02 | Yes |  |
| **Normalized copy numbers method** | | | | | | | | |
|  | Sex | | | Prognosis | Sex + prognosis | | | Breed+ prognosis |
| Multivariate analysis | 0.02 | | | 0.05 | 0.001 | | | 0.99 |
| univariate analysis | | | | | | | | |
|  | p | p adj | sig |  | p | p adj | sig |  |
| eca-miR-24 | 0.48 |  |  |  | 0.001 | 0.01 | Yes |  |
| eca-miR-125a-5p | 0.5 |  |  |  | 0.001 | 0.006 | Yes |  |
| eca-miR-127 | 0.0005 | 0.006 | Yes |  | 0.09 |  |  |  |
| eca-miR-134 | 0.003 | 0.02 | Yes |  | 0.02 | 0.018 | Yes |  |
| eca-miR-379 | 0.004 | 0.03 | Yes |  | 0.34 |  |  |  |
| eca-miR-381 | 0.94 |  |  |  | 0.08 |  |  |  |
| eca-miR-382 | 0.006 | 0.03 | Yes |  | 0.002 | 0.019 | Yes |  |
| Eca-miR-432 | 0.002 | 0.01 | Yes |  | 0.23 |  |  |  |

The first model (A) evaluates the diagnostic potential of the candidate miRNAs when comparing ES-affected [RGR (n = 19) and PGR (n = 9)] and tumor-free horses [NO (n = 19) and CTL (n = 30)] and the influence of breed and sex. The second model (C) evaluates the potential of the candidate miRNAs to predict the development of ES lesions in ES-affected horses [RGR (n = 19) vs. PGR (n = 9)]. For this model, the influence of breed and sex could not be assessed due to the small size of the PGR group (n = 9). The third model (B) evaluates the prognostic potential of the candidate miRNAs to predict new appearance of ES in so far ES free horses [NO (n = 19) vs. CTL group (n = 30)] and the influence of breed and sex. Results of both normalization methods (2^-∆∆Cq^ method and normalized copy number method) are shown. Abbreviations: CTL = Control, NO = new occurrence, RGR = regression, PGR = progression, p adj = adjusted p-value, sig = significant.
