# Supplementary figures and images for: Diagnostic and prognostic potential of eight whole blood microRNAs for equine sarcoid disease
Source: PLoS One. 2021 Dec 23;16(12):e0261076. doi: 10.1371/journal.pone.0261076 (PMC8699634; doi:10.1371/journal.pone.0261076)

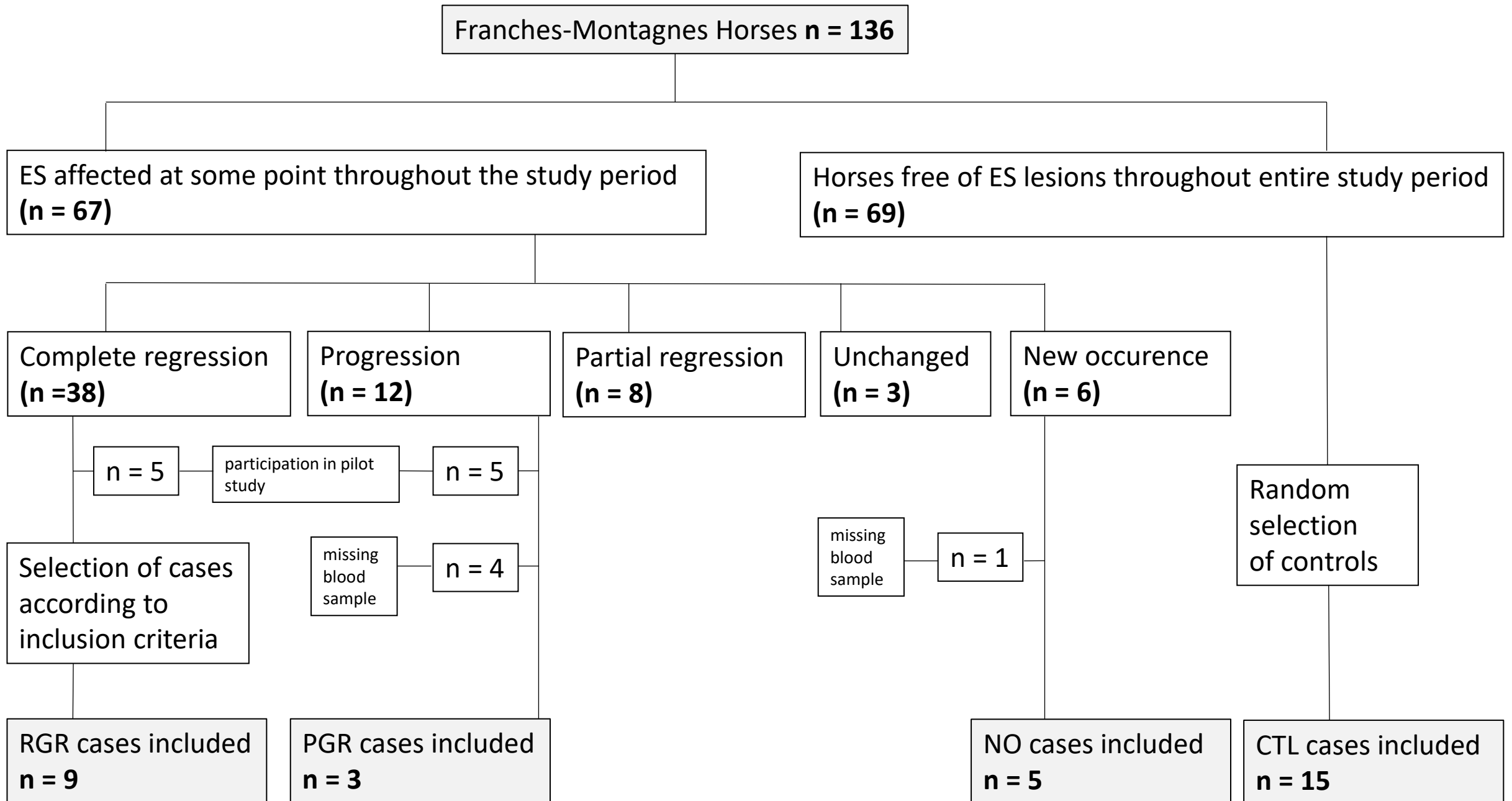

Supplement: S1 Fig — (PDF) [file pone.0261076.s007.pdf]

**A**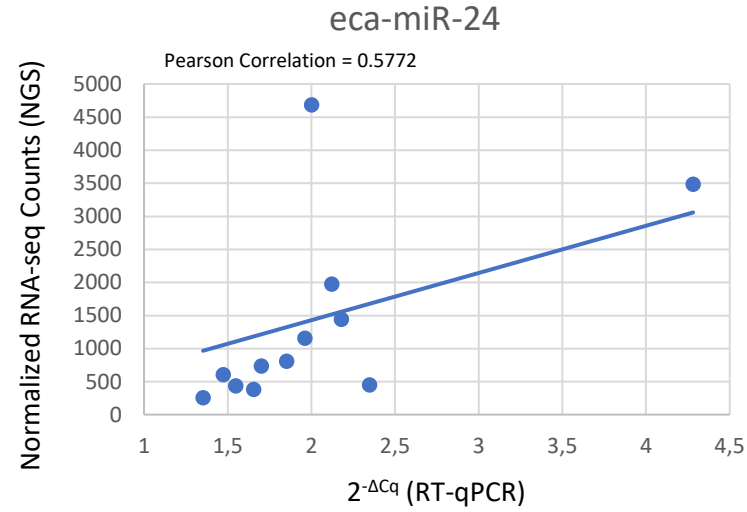**B**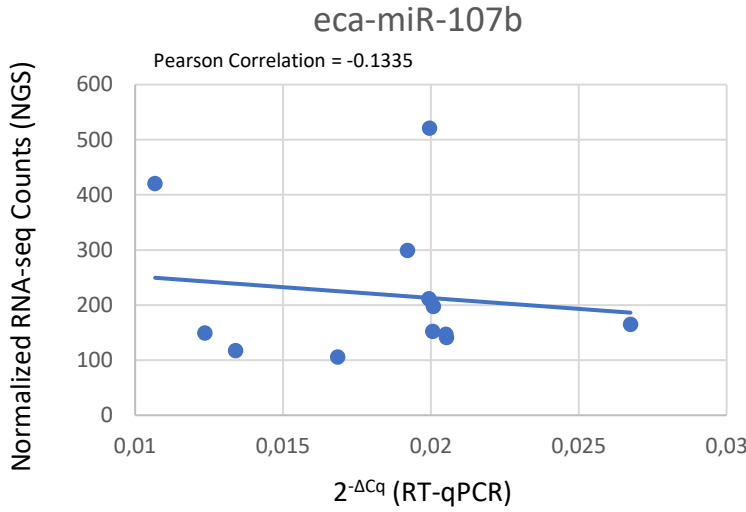**C**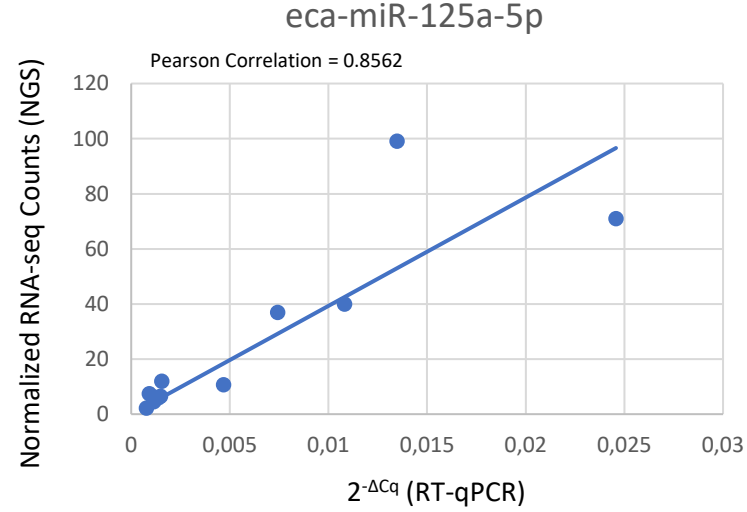**D**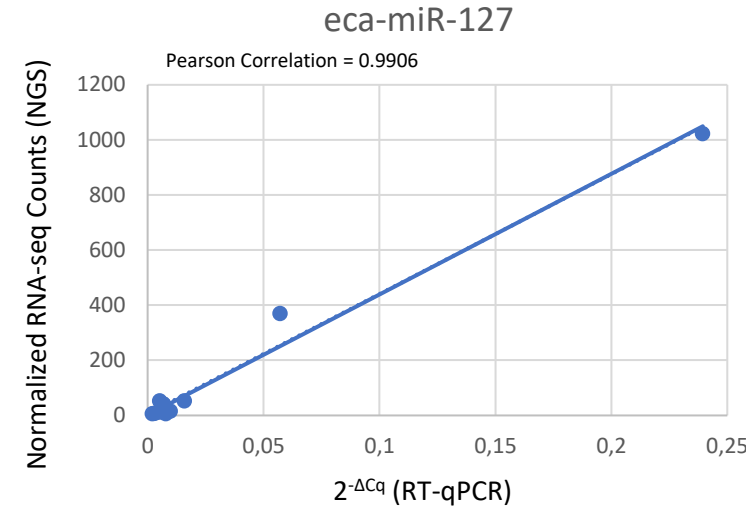**E**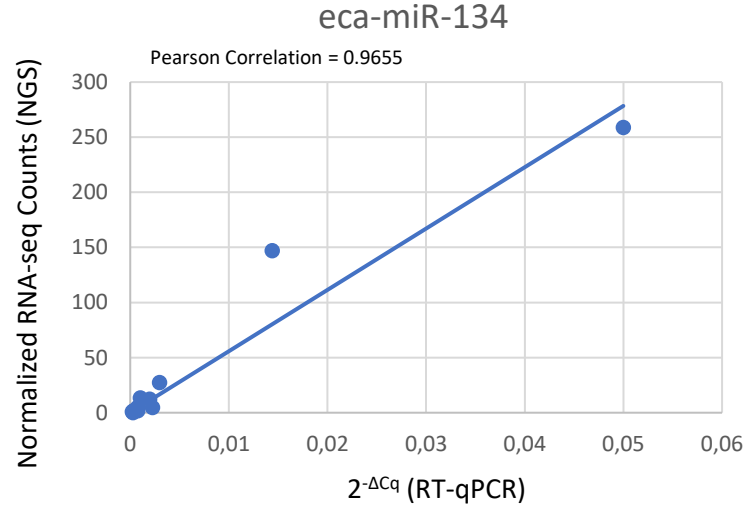**F**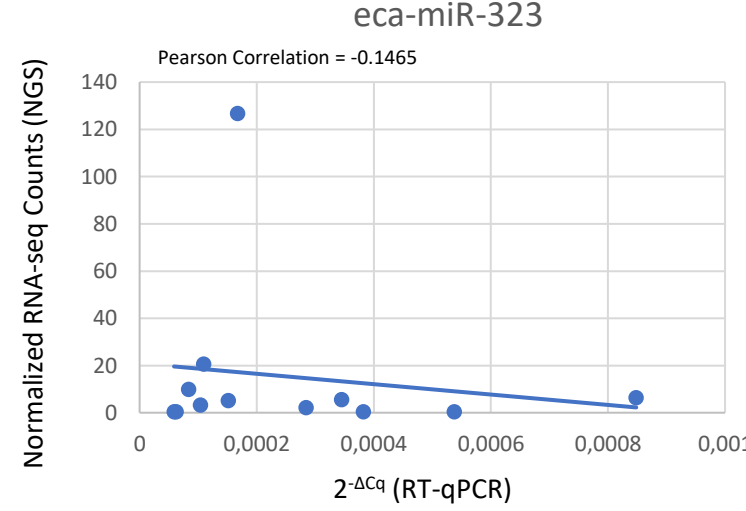**G**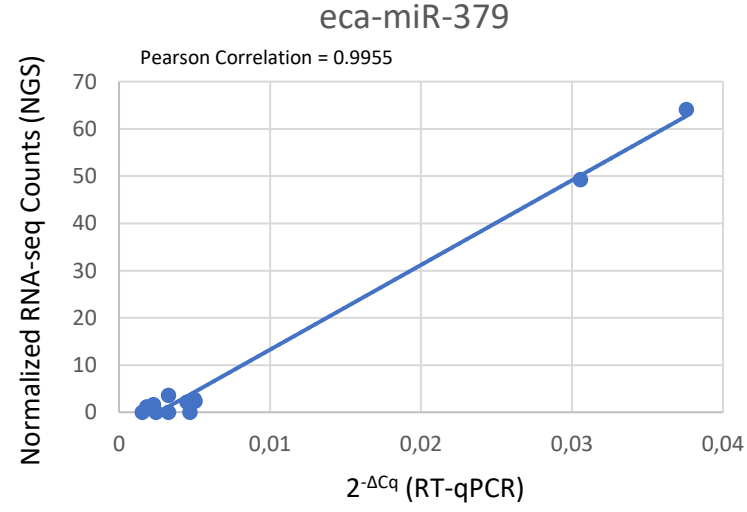**H**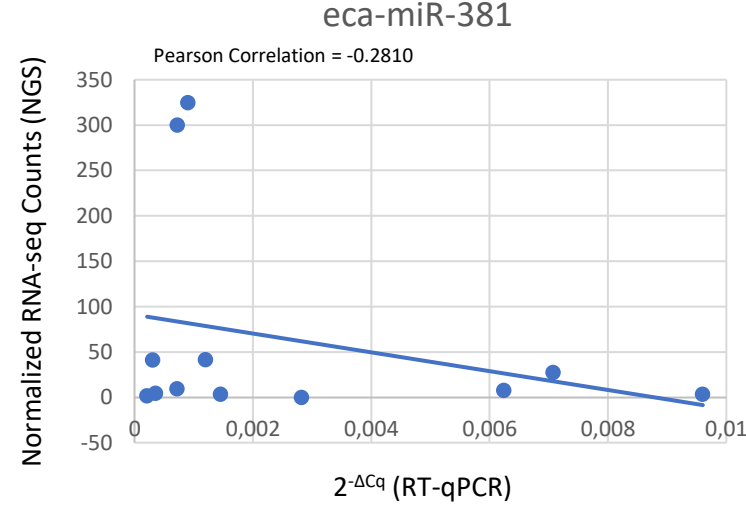**I**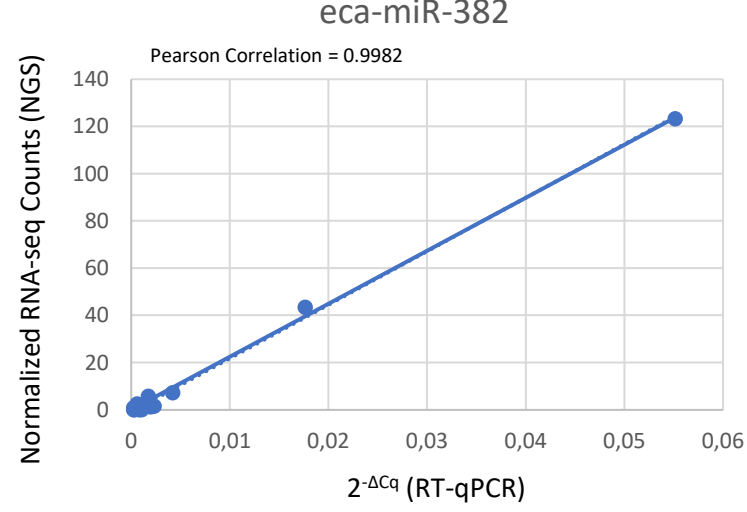**J**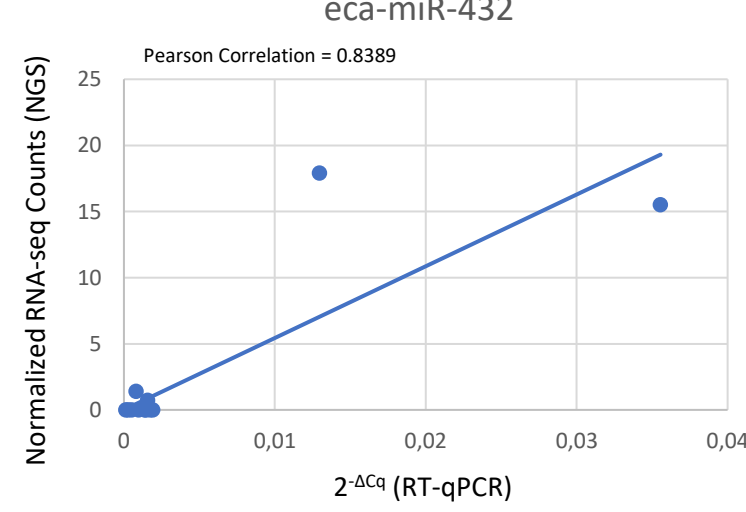

Supplement: S3 Fig — (PDF) [file pone.0261076.s009.pdf]
