# Supplementary material for: Diagnostic and prognostic potential of eight whole blood microRNAs for equine sarcoid disease
Source: PLoS One. 2021 Dec 23;16(12):e0261076. doi: 10.1371/journal.pone.0261076 (PMC8699634; doi:10.1371/journal.pone.0261076)
Supplement: S2 Fig — (PDF) [file pone.0261076.s008.pdf]

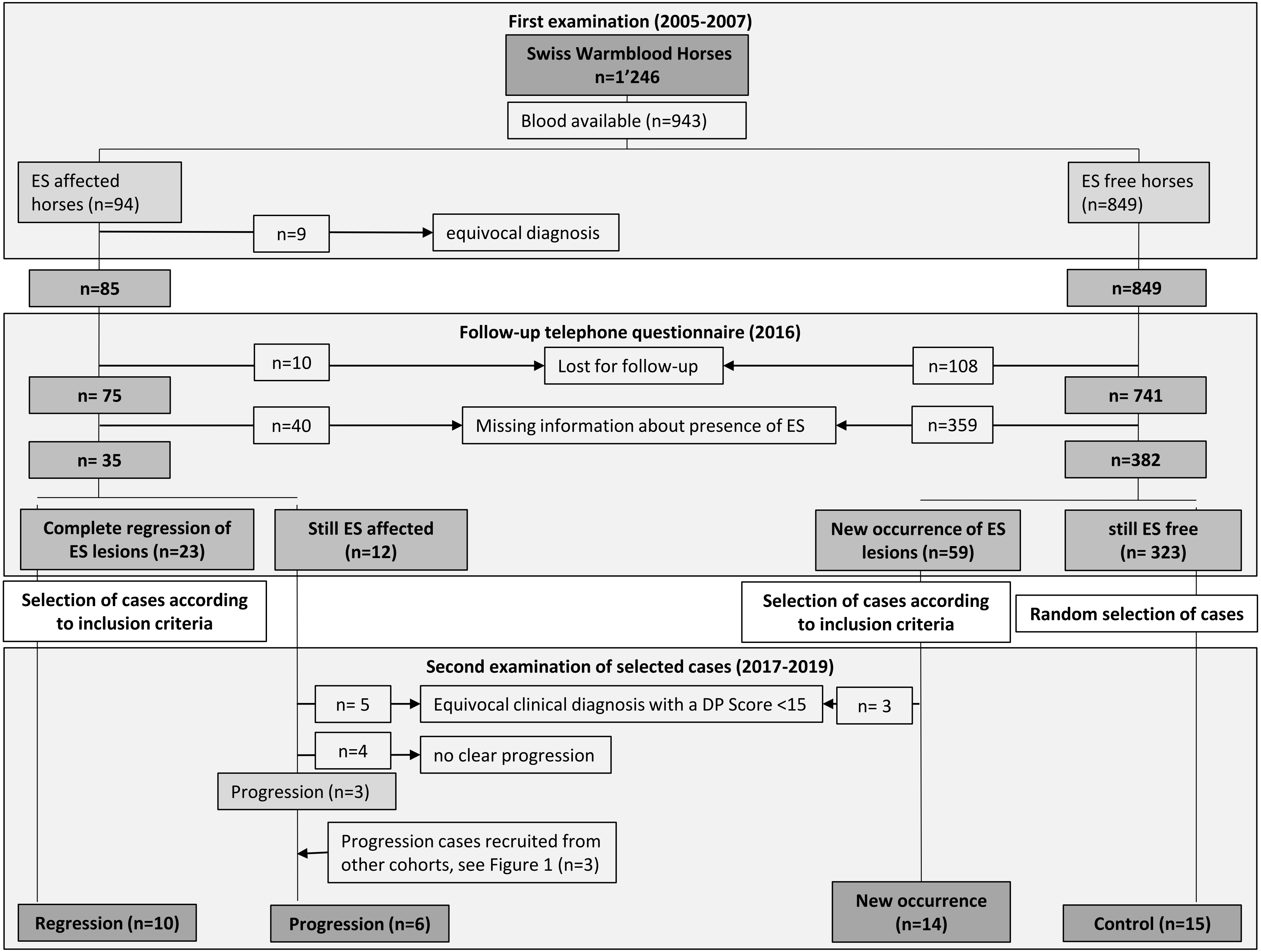
First examination (2005-2007)
Swiss Warmblood Horses n=1'246
Blood available (n=943)
ES affected horses (n=94)
ES free horses (n=849)
n=9
equivocal diagnosis
n=85
n=849
Follow-up telephone questionnaire (2016)
n=10
Lost for follow-up
n=108
n= 75
n= 741
n=40
Missing information about presence of ES
n=359
n= 35
n=382
Complete regression of ES lesions (n=23)
Still ES affected (n=12)
New occurrence of ES lesions (n=59)
still ES free (n= 323)
Selection of cases according to inclusion criteria
Selection of cases according to inclusion criteria
Random selection of cases
Second examination of selected cases (2017-2019)
n= 5
Equivocal clinical diagnosis with a DP Score <15
n= 3
n=4
no clear progression
Progression (n=3)
Progression cases recruited from other cohorts, see Figure 1 (n=3)
Regression (n=10)
Progression (n=6)
New occurrence (n=14)
Control (n=15)
